# The blushing brain: neural substrates of cheek temperature increase in response to self-observation

**DOI:** 10.1098/rspb.2024.0958

**Published:** 2024-07-17

**Authors:** Milica Nikolić, Simone di Plinio, Disa Sauter, Christian Keysers, Valeria Gazzola

**Affiliations:** ^1^ Institute for Child Development and Education, University of Amsterdam, Amsterdam 1018 WS, The Netherlands; ^2^ Department of Neuroscience, Imaging, and Clinical Sciences, D'Annunzio University of Chieti–Pescara, Pescara 66100, Italy; ^3^ Psychology Institute, University of Amsterdam, Amsterdam 1018 WS, The Netherlands; ^4^ Netherlands Institute for Neuroscience, KNAW, Amsterdam 1105 BA, The Netherlands

**Keywords:** blushing, embarrassment, self-awareness

## Abstract

Darwin proposed that blushing—the reddening of the face owing to heightened self-awareness—is ‘the most human of all expressions’. Yet, relatively little is known about the underlying mechanisms of blushing. Theories diverge on whether it is a rapid, spontaneous emotional response that does not involve reflection upon the self or whether it results from higher-order socio-cognitive processes. Investigating the neural substrates of blushing can shed light on the mental processes underlying blushing and the mechanisms involved in self-awareness. To reveal neural activity associated with blushing, 16–20 year-old participants (*n* = 40) watched pre-recorded videos of themselves (versus other people as a control condition) singing karaoke in a magnetic resonance imaging scanner. We measured participants’ cheek temperature increase—an indicator of blushing—and their brain activity. The results showed that blushing is higher when watching oneself versus others sing. Those who blushed more while watching themselves sing had, on average, higher activation in the cerebellum (lobule V) and the left paracentral lobe and exhibited more time-locked processing of the videos in early visual cortices. These findings show that blushing is associated with the activation of brain areas involved in emotional arousal, suggesting that it may occur independently of higher-order socio-cognitive processes. Our results provide new avenues for future research on self-awareness in infants and non-human animals.

## 1. Introduction

Most of us have experienced blushing—the involuntary reddening of the face that accompanies self-conscious emotions, such as embarrassment, shyness, shame and pride [1–3]. Although Darwin considered it universal and ‘the most human of all expressions’ [4], we still know relatively little about the mechanisms that cause blushing. Theories of blushing disagree on how it comes about. One framework proposes that blushing originates from higher-level cognitive processing. In this view, blushing emerges when a person reflects upon the self, considers how other people perceive them and realizes that there is a possibility of making a negative impression on others [2,4–6]. An alternative account argues that blushing is triggered independently of higher-order socio-cognitive abilities as a ‘sudden surge of alertness’ when socially exposed [7] and as a spontaneous emotional response to social exposure [8,9]. These two lines of thought reflect a broader debate between the experiential versus higher-order theories of self-awareness (e.g. [10–15]). Here, we aim to contribute to this debate by investigating the neural correlates of blushing: is blushing associated with activity in the brain regions linked to higher-order socio-cognitive abilities or those linked to emotional arousal?

Psychological theories of blushing propose that people blush when they experience heightened self-awareness and when they feel exposed or scrutinized by others [1,2,16,17]. In line with this notion, past studies have found that people blush when they perform or watch embarrassing videos of themselves in the presence of an audience [18–20], when they receive negative or overly positive feedback [3,21,22], when they disclose personal information to others [23,24] and when they are simply stared at [9]. Although these studies reveal the contexts in which blushing occurs, they do not answer the question of why blushing occurs in these contexts.

Darwin was the first to theorize about the mechanisms of blushing [4]. He wrote that it is ‘the thinking of what others think of us, which excites a blush’ (p. 325). Many scholars since Darwin agree with the notion that blushing occurs when people reflect upon the self from the perspective of others [6] and when they recognize that others may judge them negatively [2,25,26]. On this view, thus, blushing is elicited by mentalizing about others in relation to the self. However, some scholars have noted that blushing may arise as an automatic emotional response to sudden, unexpected or intense social exposure [7–9]. From that perspective, blushing is a consequence of a high level of ambivalent emotional arousal that occurs when a person feels threatened and wants to flee but, at the same time, feels the urge not to give up the social relationship [27]. Therefore, it is an open question whether higher-order socio-cognitive processes are necessary to elicit blushing. Investigating the brain regions that activate with more blushing may be informative for elucidating the mechanisms of blushing.

Surprisingly, few studies have investigated neural responses under conditions that are suited to trigger blushing. We know that self-referential processing in different domains including self- versus other-related trait adjectives, memories, emotions and movements recruits ventral, dorsal and posterior cortical midline structures [28]. We also know that exposing participants to fictional scenarios or vignettes that trigger cognitions related to self-conscious emotions recruits the medial prefrontal cortex (mPFC) (for an overview of these studies, refer [29]). To evoke an affective response, however, a realistic emotion-evoking situation that is personally relevant, rather than a hypothetical scenario, is needed [30,31]. Only three studies have measured neural responses in self-conscious emotion-evoking situations [30–32]. These studies found activation in mPFC and the ventral anterior insula—a region associated with salience and arousal, awareness of one’s emotional and body state and self-reflection. Concurrently, increased physiological arousal (i.e. skin conductance and pupil dilation) has been found in these studies. However, none of these studies measured or specifically tried to trigger the affective bodily response of blushing.

To measure the neural correlates of blushing directly, we used a paradigm that is known to induce heightened self-awareness and blushing: watching a video of oneself [18,19,33,34]. In the present study, participants watched previously recorded videos of themselves singing while their brain activity was measured with functional magnetic resonance imaging (fMRI) and cheek temperature increase was measured to reliably capture blushing [23,35]. As control stimuli, they watched another participant, matched to their singing ability, and a professional singer sang the same songs. We recruited adolescents as participants because of their heightened sensitivity to social evaluation and self-awareness [36]. To additionally probe whether individual differences in brain activity would be associated with individual differences in blushing, we recruited adolescents with varying levels of social anxiety because individuals with high social anxiety blush more [37,38].

Based on the idea that blushing occurs with heightened self-awareness, we hypothesized that participants would blush more while watching themselves sing as compared with watching others sing [18,19,33,34]. Regarding brain activity related to blushing, we sought to test two theories that make distinct predictions: if blushing is primarily linked to mentalizing about others [2,4,6], more blushing should be associated with greater activity in the brain regions associated with mentalizing (e.g. mPFC and right temporoparietal junction (rTPJ)) [39]. If blushing is independent of mentalizing and instead reflects a rapid, spontaneous emotional response to social exposure [7,9,27], more blushing should be associated with greater activity in brain areas involved in emotional arousal (e.g. anterior insula and brain stem).

### (a) Open practices statement

The present study was not pre-registered. Raw magnetic resonance imaging (MRI) data and video data cannot be shared owing to the risk of personal identification. All summary data used for the figures as well as physiological and behavioural data can be found at OSF.io: osf.io/3mhdv [40].

## 2. Methods

### (a) Participants

Sixty-three female adolescents aged 16–20 years from Amsterdam, The Netherlands, and the surrounding area took part in the study. They were recruited through advertisements on social media (Facebook and Instagram) and from the local student pool of the Faculty of Social and Behavioural Sciences, University of Amsterdam. When signing up for the study, participants did not know that they would sing karaoke in order not to bias recruitment towards participants who would feel more comfortable singing. The recruitment flyer and the information letter that participants received before visiting the laboratory revealed only that participants would take part in a social task and that they would watch some videos in an MRI scanner. Potential participants first filled in an online questionnaire on social anxiety symptoms. Those scoring high or low on social anxiety symptoms were invited to take part in the study. Sixty-three participants came to the laboratory. At the laboratory, they found out that they would sing karaoke. No participant withdrew at this point. Out of 63 participants who came to the laboratory to record the stimuli for the study, 49 participants came for the MRI study. Most of the participants who did not participate in the MRI session dropped out because they were not eligible for the scanner (e.g. wearing a permanent piercing or having an IUD). Nine participants were excluded from the fMRI analyses owing to errors during fMRI data acquisition, resulting in a final sample of 40 participants (*M*_age_ = 19.3 years, s.d. = 1.10) for the main analyses. Out of the 40 participants, one participant had missing data on the cheek temperature measure and one participant had missing data on self-reported embarrassment owing to technical errors. Participants were compensated for their travel and received 30 euros or three student credits for their participation. The study was approved by the Ethics Review Board of the Faculty of Social and Behavioural Sciences, the University of Amsterdam, under the protocol 2020-SP−11726.

We based our analyses on 40 participants in line with standards in the fMRI literature, and this affords 80% power to detect the following effects: (i) for contrasts between conditions in the fMRI analysis, with *α* = 0.001, *t*-tests, *d* = 0.66; (ii) for regressions with a subject-level predictor in fMRI analysis with *α* = 0.001, regression, *β* = 0.55; (iii) for multiple regressions using prinicipal component analysis (PCA) at *α* = 0.05, regression analysis, *f*^2^ = 0.30; and (iv) for associations between behavioural measures using correlations at *α* = 0.05, *r* = 0.37. Wherever possible, we supplement the frequentist statistics with Bayes factors to help interpret whether non-significant findings are inconclusive (3 > *BF*_10_ > 1/3) or provide evidence of absence (*BF*_10_ < 1/3, refer [41]).

### (b) Procedure

Participants came to the Behavioural Science laboratory at the University of Amsterdam twice. On the first visit, they were asked to sing karaoke while being video recorded. At the second visit, they watched videos of themselves singing as well as videos of another participant and a professional singer (whom they were led to believe to be another participant) singing while they were in an MRI scanner.

During the first visit, participants were instructed to wear a black shirt and were told that they would sing karaoke. They were positioned in front of a large screen and asked to sing while listening to the melody of the songs on headphones and seeing the lyrics on the screen. A camcorder was positioned next to the screen, which recorded the participant’s upper body and face while singing. Participants sang four songs: ‘Hello’ by Adele, ‘Let it go’ from Frozen, ‘All I want for Christmas is you’ by Mariah Kerry and ‘All the things you said’ by tATu. These songs were chosen because several music experts judged these songs to be very difficult to sing, which should ensure that participants would be embarrassed watching themselves singing them and several students judged these songs to be quite familiar to teenagers. The final set of recordings consisted of 15 video segments from the four songs, with each video segment lasting 24 s. In addition, a professional singer of around the same age as the participants was invited to the laboratory and was pre-recorded singing the same songs. The same video segments were made to be shown to our participants at the second laboratory visit. After karaoke, participants filled in several online questionnaires that were unrelated to the present study. Once videos had been recorded for a group of 10–15 participants, three video segments of each of these participants were assessed for the quality of singing by means of an online questionnaire, completed by a set of independent assessors (see electronic supplementary material for details).

On the second visit to the laboratory, participants underwent an fMRI session during which they completed the experimental task detailed below. During the experimental task in the scanner, we measured participants’ left cheek temperature continuously to assess their blushing. After the session, participants were led into another room and asked to re-watch the videos of themselves and rate how embarrassed they felt while they were watching the videos in the scanner on a scale of 0–100. At the end of the session, they were debriefed and compensated for the study.

### (c) Experimental task

While in the MRI scanner, participants were instructed that they would watch videos of themselves and other participants singing karaoke. To increase embarrassment, they were additionally told that, at the same time, other participants were watching their videos in scanners close by. Participants watched 15 video segments of themselves singing and, as a control, 15 corresponding video segments of another participant who had been assessed as the most similar in singing quality to the current participant. To control for the possibility that a participant would experience vicarious embarrassment for the other participant who sang similarly (badly) to themselves (based on the findings that people can blush empathetically [42]), we introduced an additional control condition: participants watched 15 corresponding video segments of a professional singer, whom we pre-recorded and introduced to participants as ‘another participant of the study’. As the professional singer sang considerably better, which we confirmed by ratings of independent assessors (for details, see the electronic supplementary material), we did not expect these videos to trigger vicarious embarrassment.

The fMRI paradigm involved three sessions with five trials each. Each trial consisted of the same video segment sung by the participant, another participant and the professional (i.e. three video segments per trial). Stimuli within each trial and trials within each session were randomized in advance and shown to participants in a fixed order. This choice was made to allow for inter-subject correlation (ISC) analyses, which require a fixed presentation order across participants. The three sessions were shown to participants in a random order to minimize the risk of order effects. Between each video segment, there was a 12 s inter-trial period during which the participant saw a black screen.

### (d) Behavioural and physiological measures

#### (i) Social anxiety symptoms

During recruitment, we asked potential participants to fill in the brief version of the Social Phobia and Anxiety Inventory (SPAI−18 [43]), a validated and reliable 18-item questionnaire constructed from the original SPAI−18 to assess a broad range of clinically relevant social anxiety symptoms [43]. Adolescents scoring above 48 (the clinical cut-off score in The Netherlands [43]) and below 27.80 (mean score for non-clinical Dutch people [43]) were invited to participate in our study as adolescents with high and low levels of social anxiety, respectively. Internal reliability of the SPAI−18 in our adolescent sample was excellent, Cronbach’s *α* = 0.97.

#### (ii) Cheek temperature

We indexed blushing as the increase in cheek temperature from the 12 s period before the stimulus was shown to the 24 s period during which the video was shown to the participant. Using the increase in cheek temperature within 30 s of the onset of the emotionally charged social event as a measure of blushing has been validated in past studies [33,44]. As compared to blood flow measures that are often used to capture blushing, the measurement of temperature is less prone to artefacts and results in a stable signal that can capture slow and large changes in blushing [35].

Cheek temperature was captured with the Biopac MRI-compatible, TSD202 series thermistor (temperature) transducer validated for human use in the scanner. The temperature transducer is a fast-response temperature probe appropriate for use in bodily locations where temperature changes rapidly, with a response time of 0.6 s and dimensions: 1.7 mm (dia) × 5 mm (long). For our study, we attached the temperature probe over the left cheekbone of participants with Biopac-recommended tape.

#### (iii) Self-reported embarrassment

After the session in the scanner, participants re-watched the videos of their own performance that they had previously watched in the scanner. Participants were asked on a computer screen, for each video, how embarrassed they felt while watching the video in the scanner on a visual scale from 0 = not at all to 100 = extremely embarrassed. Participants used a mouse and a slider on the visual scale on the screen to give a response. Participants were first shown an example of the visual scale and instructed how to use the slide.

### (e) Analysis of behavioural and physiological data

The effect of the experimental condition on blushing responses was assessed using a mixed-effects regression model. The dependent variable was the average blushing response for each participant, for each experimental condition, calculated as the temperature increase (i.e. a difference score) from the 12 s baseline (inter-trial interval) to the 24 s trial (during the trial, that is, video watching), averaged across trials of the same condition. The experimental condition was added as a categorical, within-subject factor including three levels: Self, Other and Professional. A random intercept was included at the subject level. Thus, the Wilkinson notation for the model was ‘blushing~condition + (1|subject)’. Linear tests were performed within the model to check all the combinations of effects and to report statistics of interest. The model was checked for normality of residuals’ distributions, for problematic collinearity and homoskedasticity.

A Bayesian repeated-measures ANOVA was run in parallel to estimate posterior odds and Bayes factor quantifying evidence for the alternative hypothesis relative to the null hypothesis. The model used for the analysis (including the term Condition) was compared with the model containing the grand mean and the random factors (null model). Contrasts in the Bayesian repeated-measures ANOVA were corrected for multiple testing by fixing at 0.5 the prior probability that the null hypothesis holds across all the comparisons [45]. Q–Q plots indicated residuals to be well-behaved, and we therefore continued with the parametric estimation. Individual comparisons were based on default *t*-tests with a Cauchy prior. As temperature scores were not normally distributed (Shapiro–Wilk < 0.911, *p* < 0.004), we used non-parametric Wilcoxon signed-rank tests. Behavioural analyses including mixed-effects modelling were performed in Matlab [46]. Bayesian testing was performed using JASP [47].

In addition, we ran the same mixed-effect model (and follow-up Bayesian repeated-measures ANOVA) with the addition of social anxiety scores as a predictor and an interaction between condition and social anxiety to account for the possibility that social anxiety alone or in combination with the condition accounted for individual differences in blushing, which was not the case. We report these analyses in the electronic supplementary material.

### (f) Functional magnetic resonance imaging data pre-processing

Standard (f)MRI pre-processing steps were applied using the software Analysis of Functional NeuroImages (AFNI [48]). The functional images were despiked, deobliqued and time-shifted to match the acquisition timing across slices. Images were then motion-corrected using a six-parameter rigid body realignment and aligned to the T1, which was skull-stripped and aligned to the Montreal Neurological Institute (MNI) template through nonlinear warping interpolation. The normalization parameters obtained through this step were then applied to the functional images. During pre-processing, motion parameters were saved to use as nuisance regressors in further analyses. Spatial smoothing was applied to fMRI images using a Gaussian kernel of 6 mm full width at half maximum. Finally, time series were scaled to have an average value of 100 and produce ‘percent signal change’, which is a more interpretable measure [49]. The pre-processing diagnostic was performed using AFNI built-in quality check programmes and through the direct examination of the pre-processed images.

### (g) Analysis of magnetic resonance imaging data

#### (i) Neural correlates of cheek temperature changes

To capture neural substrates associated with trial-by-trial variability in cheek temperature changes independently of the experimental condition, we performed an analysis in which the single-subject regression model was implemented using two regressors. The first regressor, *trials*, modelled the average activity across all trials, using a box-car regressor capturing the duration of all trials, independently of condition. The second regressor, *cheek temperature change*, was an amplitude-modulated version of the first one, with the same onsets and duration, but with the amplitude modulated by the average cheek temperature change response of each trial. We then performed a group-level *t*‐test against zero using the coefficients of cheek temperature change as the dependent variable.

#### (ii) Analysis of task-evoked activity

First, we used general linear modelling at the single-subject level to estimate average differences in brain activity across experimental conditions. Single-subject stimulus regressors were created by convolving the stimulus onset with haemodynamic-response block functions with a fixed duration corresponding to the duration of the trials (24 s) and with a fixed amplitude of 1. A regression model was applied to estimate single-subject activation maps for each experimental condition (Self, Other and Professional). The group analysis was performed using a linear mixed-effects model through the function 3dLMEr in AFNI. In the group analysis, input datasets were the single-subject activation coefficient (*β*) maps in each condition. Independent variables included the categorical factor Condition with three levels and a continuous regressor of interest Blushing, representing the average level of temperature increase for each subject in each condition. A random intercept was added at the subject level.

#### (iii) Analysis of inter-subject correlations

We analysed cross-subject similarities in dynamic brain activity using ISC methods. ISC allows for the measurement of the extent of blood oxygen level-dependent (BOLD) response similarity within a group of subjects that were scanned while perceiving the same type of stimulation and for identification of brain activity that is systematically associated with the common stimulus [50]. Additional pre-processing steps included high-pass filtering (>0.01 Hz) and nuisance regression, which were applied in a single analysis step in agreement with current guidelines [51]. Noise regressors included motion regressors, white matter and cerebrospinal fluid signals extracted during antecedent pre-processing steps. The similarity in brain activity for each pair of subjects was computed by calculating the Pearson product–moment correlation coefficients between the pre-processed individual time series. Correlation maps were calculated separately for each experimental condition after re-ordering and concatenating trials of each condition for each participant so that correlations were calculated from BOLD signals evoked by the same movie. The first 12 volumes, corresponding to 8 s, were trimmed from the onset of each trial to remove the general effect of the initial transient [50]. The resulting correlation maps were transformed using Fisher-*z* transformation and analysed at the group level using the program 3dISC in AFNI [48], which uses voxel-wise linear mixed-effects modelling [52]. ISC group maps were calculated for simple conditions (Self, Other and Professional) and linear contrasts of interest (Self–Other, Self–Professional and Other–Professional). In addition, a further ISC model was built in which the continuous regressor Blushing was added, after centring it around zero, to locate regions in which similarity in patterns of brain activity was associated with similarity in average blushing responses.

For all the group-level fMRI analyses, significance was tested using cluster-level thresholding calculated by simulating noise volumes through mixed modelling of the autocorrelation structure, which has been shown to give accurate control of the false-positive rate [53].

## 3. Results

### (a) The effects of condition on blushing: watching oneself sing is associated with increases in blushing

**Figure 1.**
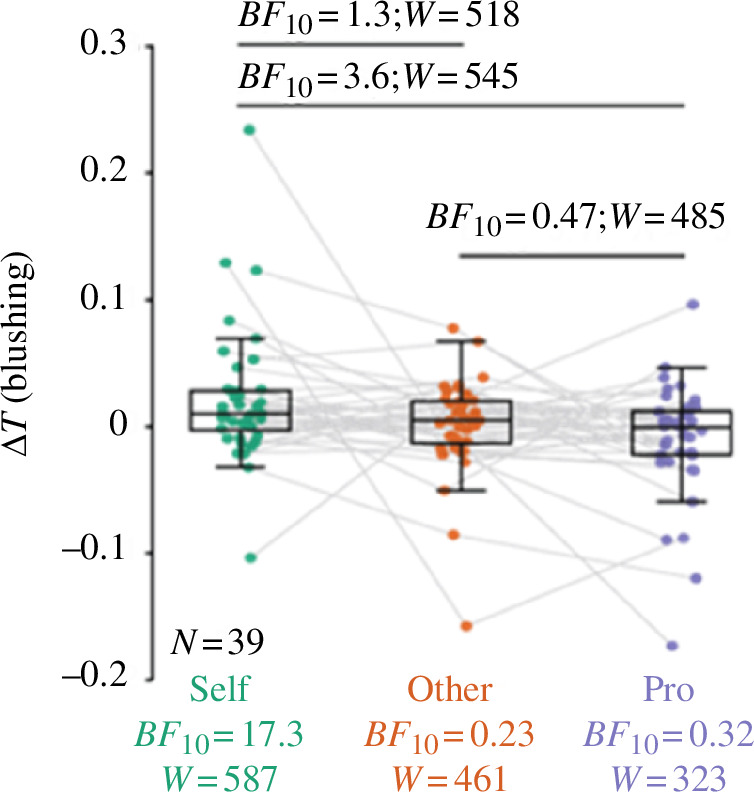
Effects of the experimental condition on blushing (cheek temperature increase). Δ*T* = difference in temperature (in ℃) between the 12 s prior to movie onset and the 24 s of the movie. This difference is used as a proxy for blushing. Each dot represents the average Δ*T* over all trials of each individual, computed separately for the Self (green), Other (orange) and Professional (purple) conditions. Bayesian values resulting from the *t*‐test versus zero are indicated in coloured text under each condition; values from the paired *t*-tests are indicated in black over the boxes. Non-parametric Wilcoxon signed-rank tests were used because the Δ*T* was not normally distributed. The Wilcoxon values are also indicated as *W*. The boxes represent the first, second and third quartiles; the whiskers represent the minimum and maximum values within the 1.5 interquartile range.

Mixed-effects models showed a significant effect of the experimental condition on blushing, *F*_(2,111)_ = 4.7, *p* = 0.010. The Self-viewing condition elicited increases in cheek temperature (figure 1, green), whereas in the Other-viewing condition, there was relatively no modulation of the temperature (figure 1, orange). In the Professional-viewing condition, a slight decrease in the temperature from baseline to the period of watching the video was observed (figure 1, purple). These results were confirmed using linear tests for each individual condition against baseline within the mixed-effects model: blushing was significantly higher in the Self condition compared with the average (*β* = 0.022, *p* = 0.0049, *t*_(39)_ = 2.9), but it was not significantly different from average in the Professional condition (*β* = −0.011, *p* = 0.16, *t*_(39)_ = −1.4) or in the Other condition (*β* = 0.0009, *p* = 0.90, *t*_(39)_ = 0.1). We further performed paired linear tests between each condition pair within the mixed-effects model. These tests showed that the blushing responses in the Self condition were significantly higher than in the Professional condition (*β* = 0.033, *p* = 0.003, *t*_(39)_ = 3.0) and not significantly different from the Other condition (*β* = 0.021, *p* = 0.055, *t*_(39)_ = 1.9). The contrast between the Other and Professional conditions was not significant: *β* = −0.012, *p* = 0.27, *t*_(39)_ = −1.1. These results are illustrated in figure 1. A Bayesian repeated-measures ANOVA confirmed that the experimental conditions differentially influenced blushing (*BF*_incl_ = 5.82). Follow-up one-sample *t*-tests showed strong evidence for the Self condition to elicit a difference in blushing of greater than zero (*BF*_10_ = 17.3), whereas the other conditions remained closer to zero (Other *BF*_10_ = 0.23; Professional *BF*_10_ = 0.32). The Bayesian paired *t*-tests directly comparing conditions with each other, supported a difference between the Self and Professional conditions (*BF*_10_ = 3.6) but was anecdotal for the other comparisons (figure 1).

### (b) Functional magnetic resonance imaging preliminary analyses

#### (i) Seeing oneself sing is associated with increased activity in regions associated with arousal and salience but not the default mode network

**Figure 2.**
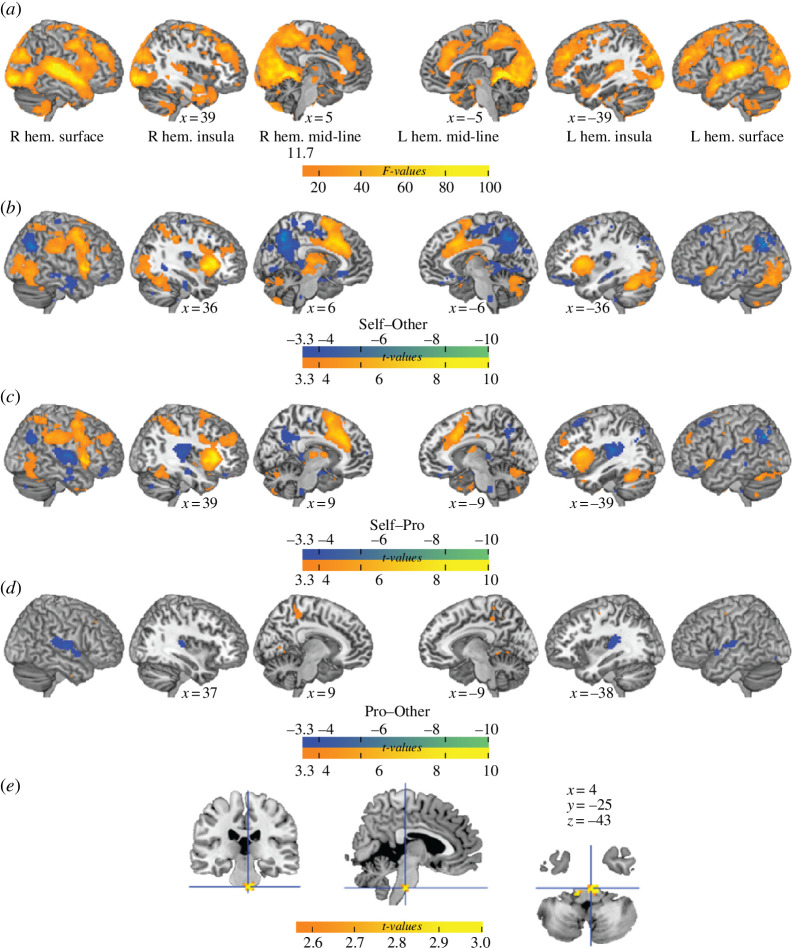
Effects of experimental condition on brain activity and brain activity related to cheek temperature changes. (a) Main effect of watching someone singing (*F* = 11.73, *k* = 40, *p* < 0.001). (*b–d*) *t*‐test comparing the effect of watching different singers (*t* = 3.29, *k* = 40, *p* < 0.001). Warm colours indicate positive values and blue indicates negative values. Contrasts overlaid on the ch2better template rendered in MRIcron (https://www.nitrc.org/projects/mricron) with a search depth of 8 voxels. (*e*) Associations between task activity and cheek temperature independent of condition, cluster corrected. Peak coordinates: [5 −25 −43]; *ɑ* < 0.01 (cluster corrected; *k* = 50), a critical threshold for the *t*-statistic = 2.57.

The analysis of task-dependent activity revealed that, on average, viewing individuals sing engaged visual/occipital and auditory/temporal regions as well as the posterior and anterior regions of the insular cortex (figure 2*a*). The contrast between conditions (figure 2*b–d*; electronic supplementary material, table S2) was investigated to better understand condition-specific patterns of activity, and in particular, to isolate activity that was not simply triggered by viewing and hearing someone sing, but specifically, by viewing *oneself* sing. The Self condition (compared with both Other and Professional) was, on average, associated with *increased* evoked activity in regions associated with emotional arousal, salience and attention (anterior insula, mid-cingulate cortex and dorsolateral prefrontal cortex), as well as with reduced BOLD signal in default mode regions such as the medial prefrontal cortex, the posterior cingulate cortex and the inferior parietal lobule. In addition, the Professional condition elicited more activity in auditory regions.

#### (ii) Trial-by-trial differences in blushing across conditions are associated with cranial nerve and raphe nuclei activity

Examining the neural correlates of trial-by-trial variance in cheek temperature across all trials, regardless of condition, revealed that a cluster was positively associated with the amplitude of cheek temperature at the MNI coordinates [7, −22, −41], as shown in figure 2*e*. This cluster encompassed cranial nerve nuclei (VII and V) and the raphe nucleus in the mesencephalon (figure 2*c*).

### (c) Functional magnetic resonance imaging main analyses: brain activity related to blushing

#### (i) Participants who blushed more showed, on average, stronger activity in the cerebellum and paracentral lobule

**Figure 3.**
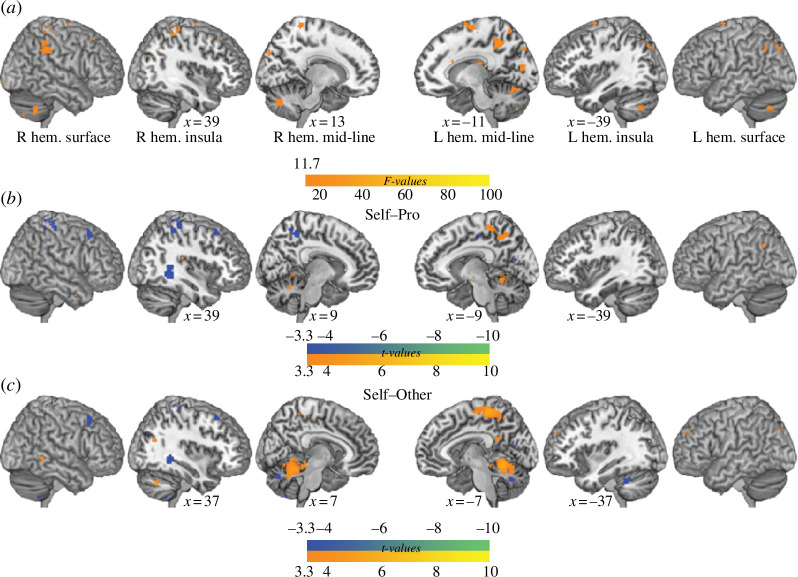
Associations between task activity and individual differences in blushing as indexed by cheek temperature increase. (*a*) Regions with parameter estimates for watching singing associated with individual differences in cheek temperature changes (*F* = 11.7, *k* = 40, *p* = 0.001). (*b–c*) Regions with significant differences in association with individual differences in cheek temperature change as a function of the singer (*t* = 3.29, *k* = 40, *p* < 0.001).

We found a positive association between individual differences in blushing and brain activity in medial superior regions of the cerebellum (lobule V) and in the left paracentral lobule (figure 3, orange; table 1). Negative associations were found in the right fusiform and angular gyri (figure 3, blue; table 1). By studying the Blushing-by-Condition interaction, we found that the effect in the cerebellum (peak coordinates: [20, −74, −32], cluster size = 176 voxels) was stronger in the Self than in the Other or Professional condition, and the effect in the left paracentral lobule (peak coordinates: [−8 −50 47], cluster size = 73 voxels) was stronger in the Self than in the Professional condition.

**Table 1.** Associations between blushing and brain activity.

*N*_vox_	regions	*β*_MEAN_	*X*_PEAK_	*Y*_PEAK_	*Z*_PEAK_	*β*_PEAK_
**activity map: self condition**						
10 676	LR-MFG, LRPreCuneus, LR-Calcarine Sulcus, LR-LG, LR-Cuneus, LRSOG, LRMOG, LR-–FG	−0.4169	10.5	76.5	−10.5	−1.7668
6114	R-STG, R-MTG, R-IFGo, R-Ins, R-PCG, R-IFGt, R-TP, R-ITG, R-IOG	0.4299	−34.5	94.5	−7.5	1.8291
3110	L-STG, L-MTG, L-IFGo, L-Ins L-PCG, L-IFGt, L-TP, L-ITG, L-IOG	0.4387	28.5	94.5	−10.5	1.5792
568	L-Cerebellum (VIII, Crus1, Crus2, VII), R-Cerebellum (VIII, Crus2, VII)	0.2906	31.5	64.5	−58.5	0.6566
241	LR-SMA	0.3198	−4.5	−10.5	70.5	0.8013
157	R-Cerebellum (VIII, Crus2, VII)	−0.2855	−40.5	40.5	−49.5	−0.4452
137	LR–MCC	0.1346	−1.5	−16.5	31.5	0.2362
109	R-Caudate, R-Thalamus	−0.0613	−19.5	25.5	22.5	−0.0974
85	R-IFGo, R-OG	−0.6159	−43.5	−52.5	−16.5	−0.918
84	L-AG	−0.1711	52.5	70.5	28.5	−0.258
66	L-IFGo, L-IFGt	0.1991	46.5	−10.5	28.5	0.2716
61	L-mSFG	−0.1390	1.5	−34.5	46.5	−0.1864
**activity map: other condition**						
6341	LR-MFG, LR-PreCuneus, LR-Calcarine Sulcus, LR-LG, LR-Cuneus, LR-SOG, LR-MOG, LR-FG	−0.5169	10.5	76.5	−10.5	−1.6409
3302	R-STG, R-MTG, R-TP, R-IOG, R-ITG, R-IFGt, R-IFGo, R-Hipp	0.4921	−25.5	97.5	−7.5	1.5863
1971	L-STG, L-MTG, L-TP, L-MOG, L-IOG, L-Hipp	0.4835	67.5	10.5	7.5	1.456
863	L-MFG, L-SFG	−0.165	34.5	−46.5	37.5	−0.3797
743	LR-ACC, L-mSFG, LR-MCC	−0.1608	1.5	−25.5	31.5	−0.3726
378	R-MFG, R-SFG, R-IFGt	−0.2042	−34.5	−58.5	25.5	−0.4397
248	R-SFG, R-MFG	−0.1159	−31.5	−10.5	64.5	−0.2569
241	L-Cerebellum (VIII, Crus2, VII)	0.2331	28.5	64.5	−58.5	0.4494
129	R-Cerebellum (VIII)	−0.2773	−40.5	46.5	−55.5	−0.4538
96	R-PCG	0.3213	−55.5	−1.5	49.5	0.703
66	R-Cerebellum (VIII, Crus2, VII)	0.1719	−10.5	79.5	−46.5	0.2329
56	R-Putamen, R-Pallidum	0.092	−22.5	−4.5	4.5	0.1309
44	L-Putamen, L-Pallidum	0.0675	22.5	−1.5	7.5	0.0984
42	L-Caudate	−0.1134	1.5	−22.5	−1.5	−0.1535
**activity map: professional condition**						
11 103	LR-MFG, LR-Calcarine Sulcus, LR-PreCuneus, LR-Cuneus, LR-LG, LR-SFG, LR-SOG, LR-MCC, LR-ACC	−0.3943	10.5	76.5	−10.5	−1.7495
3849	R-STG, R-MTG, R-IOG, R-TP, R-ITG, R-PCG, R-IFGt, R-IFGo, R-MOG	0.4979	−67.5	7.5	7.5	1.8147
2368	L-STG, L-MTG, L-MOG, L-TP, L-IOG, L-PostCG, L-SMG	0.5221	67.5	19.5	13.5	1.8423
311	L-Cerebellum (VIII, Crus2, VII)	0.2587	28.5	64.5	−58.5	0.5522
236	R-Cerebellum (VIII, Crus2, VII)	−0.2476	−55.5	46.5	−40.5	−0.5164
131	L-Putamen, L-Pallidum	0.0858	22.5	−4.5	4.5	0.1591
59	R-Cerebellum (VIII, VII)	0.179	−22.5	67.5	−52.5	0.2623
54	L-Caudate	−0.1052	1.5	−19.5	−1.5	−0.1523
49	R-vMCC	−0.0506	−19.5	1.5	28.5	−0.0819
48	R-PostCG, R-SMG	−0.1034	−55.5	7.5	28.5	−0.1416

ACC, anterior cingulate gyrus; AG, angular gyrus; FG, fusiform gyrus; Hipp, hippocampus; IFGo, inferior frontal gyrus pars opercularis; IFGt, inferior frontal gyrus pars triangularis; Ins, insular cortex; IOG, inferior occipital gyrus; ITG, inferior Temporal Gyrus; L, left; LG, lingual gyrus; LR, left and right; MCC, middle cingulate cortex; MFG, middle frontal gyrus; MOG, middle occipital gyrus; mSFG, medial superior frontal gyrus; OG, orbital gyrus; PCG, pre-central gyrus; PostCG, post-central gyrus; R, right; SMA, supplementary motor area; SMG, supramarginal gyrus; SOG, superior occipital gyrus; TP, temporal pole; vMCC, ventral middle cingulate cortex.

In contrast to the individual differences in blushing for which effect sizes suffice to survive a correction for a whole brain analysis, effects of anxiety and self-report of embarrassment on brain activity were too subtle to survive a whole brain correction (but see the electronic supplementary material for principal component regression analyses with social anxiety and embarrassment).

#### (ii) Participants who blushed more showed higher ISC in early visual cortices

ISC analyses were performed to identify networks that represent the content of the observed videos, based on the logic that activity synchronizes across participants only if the activity is time-locked to the content of the stimuli [50]. ISC maps for the three experimental conditions of the task and the contrasts between the conditions are reported in the electronic supplementary material, figure S3.

**Figure 4.**
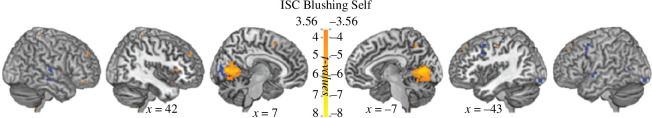
Inter-subject correlations. Regions with individual differences in inter-subject correlation that are significantly associated with individual differences in blushing (i.e. cheek temperature changes) in the Self condition. Warm colours indicate positive values and blue indicates negative values. Note that the negative correlations do not survive the cluster size correction of *k* = 40. Clusters overlaid on the ch2better template rendered in MRIcron (https://www.nitrc.org/projects/mricron) with a search depth of 8 voxels.

When investigating the association between ISCs and individual differences in blushing, we detected three maps that all included the early visual brain regions, but that slightly differed across the experimental conditions. In the Self condition, blushing was positively associated with ISC in primary (BA 17) and secondary (BA 19) visual cortices (figure 4; for other conditions, see the electronic supplementary material, figure S3 and electronic supplementary material, table S3).

## 4. Discussion

To examine neural correlates of blushing, we exposed participants to pre-recorded videos of themselves singing karaoke while measuring their physiological blushing and brain activity. We found that blushing is higher when seeing oneself sing than when seeing others sing. Individuals who blushed more while watching themselves sing had, on average, higher activation in the cerebellum (lobule V) and left paracentral lobe and showed more time-locked processing of the movies in early visual cortices. These findings suggest that blushing may be triggered by self-related processing and that it is associated with the activation of brain areas involved in emotional arousal and attention to self-relevant stimuli.

Blushing was more strongly induced by watching a video of oneself sing compared with watching videos of others sing. This suggests that blushing may be most strongly evoked in response to self-related stimuli. This aligns with the notion that blushing typically occurs when the self is exposed [2,54]. Interestingly, the direct comparison between the experimental conditions revealed that adolescents, on average, blushed more while watching themselves sing compared with watching the professional sing. However, we found only anecdotal evidence that blushing was more intense while watching oneself versus another participant sing. Our participants sang, on average, significantly worse than the professional singer. Therefore, a modest difference between the Self and Other conditions may indicate that watching other participants sing might have, at least in some participants, evoked blushing to a certain degree. This may reflect so-called empathic blushing [42] that may occur in response to ‘similar others’. As participants were matched on their quality of singing, seeing someone else sing equally badly might, thus, have evoked empathy reflected in blushing in some cases. This was not the case, however, when watching the professional singer sing as her singing was of a high quality.

We also found that, on average, participants who blushed more in response to watching themselves sing had stronger activations in the cerebellum (lobule V) and left paracentral lobule. Although the cerebellum is classically known for controlling motor behaviours, it has also been found to be involved in sensorimotor action observation and mirroring [55–58] and in auditory processing [59]. Moreover, accumulating empirical evidence suggests that the cerebellum, including lobule V, plays a crucial role in emotion processing [60]. Thus, increased activity in lobule V of the cerebellum that occurred with more blushing may imply that individuals who blushed more were engaged in sensory–motor matching and were more emotionally engaged. The activation in the mentalizing brain areas was conspicuously absent from the analysis examining the association of BOLD signals with trial-by-trial variation in blushing or individual differences in blushing. These findings, thus, provide no evidence for theories suggesting that mentalizing is key to triggering blushing in self-conscious situations.

We also found that participants who blushed more while watching themselves sing showed more time-locked processing of the movies in early visual cortices and that blushing was related to the activation of visual associative areas. These results suggest that participants who blushed might have also processed the visual stimulus more deeply [61] and were more emotionally aroused by these stimuli [62]. Emotional arousal owing to the presentation of self-relevant stimuli may have heightened subjects' attention and sensitivity to the stimuli [63,64]. Taken together, these results are in line with recent proposals suggesting that blushing might be triggered by a sudden surge of alertness when socially exposed. In this view, blushing results from pre-reflective emotional processes rather than from mentalizing [7–9,65].

Further evidence for the suggestion that blushing reflects pre-reflective arousal rather than more conscious mentalizing processes lies in the dissociation between blushing and consciously reported embarrassment and social anxiety. The degree of blushing was not associated with the levels of self-reported embarrassment that participants experienced while watching themselves sing nor with self-reported social anxiety symptoms.

Trial-by-trial changes in cheek temperature across conditions were associated with higher activity in the mesencephalon (raphe nuclei and the cranial nerve nuclei V and VII). Animal studies have shown that the raphe nuclei are responsible for body temperature regulation [66] and cranial nerve nuclei V carry temperature sensations from the face [67]. Our results thus align with previous research in animals showing that the mesencephalon, jointly with the hypothalamus, comprises the thermoregulatory centre in the brain (68).

Although watching oneself (versus others) sing was associated with more blushing on the physiological level, on the brain level, it was associated with activation in regions associated with emotional arousal and saliency (anterior insula, mid-cingulate cortex and dorsolateral prefrontal cortex) and increased stimulus processing (as proxied using ISC) in early visual regions. In contrast, it led to reduced activation in the default mode network (medial prefrontal cortex, the posterior cingulate cortex and the inferior parietal lobule). The finding that self-observation is related to activation in regions associated with emotional arousal is in line with studies that used personally relevant situations to evoke self-conscious emotional arousal (e.g. [30]). Unlike earlier studies, however, we found reduced activity in the default network and the mPFC specifically. This may be owing to the focus on external visual stimuli in our study, which may decrease activity in the mPFC [69].

In addition to exploring the neural correlates of blushing, our study provides insights into self-referential processing. In past studies, participants were asked to determine how well certain traits apply to themselves compared with others [28], which probes conscious decision-making and recruits a mentalizing network including the TPJ, precuneus and medial-prefrontal cortices anterior to the corpus callosum [70]. Our karaoke paradigm also triggers self-referential processing, as evidenced by medium-sized differences between Self versus Others in the brain and physiological responding, but does so in a more self-critical, negatively valanced context and without probing conscious decision-making. This is evident from the embarrassment scores of our participants, and the different brain networks engaged in this task as compared with those elicited by traditional paradigms (electronic supplementary material, figure S4). Combining these different approaches to probe self-referential processing in developmental studies and clinical populations may provide valuable insights into these different facets of self-referential processing.

Our study has several limitations that should be considered when interpreting these results. First, we indexed blushing as an increase in the cheek temperature, but blood flow measures may provide additional information about blushing. Unlike slower temperature changes, blood flow changes quickly and changes can be captured within a few seconds [35]. Future studies should thus implement an MRI-compatible plethysmograph when measuring blushing in the scanner. Second, whether the neural activity we found to be associated with blushing while witnessing oneself sing generalizes to other blushing-eliciting situations remains to be determined. Third, our analyses were not pre-registered and although we hypothesized that blushing may be associated with activity in the brain regions either involved in mentalizing or in emotional arousal, we did not expect to see the association with the activity in cerebellum and visual cortex specifically. Therefore, our findings call for pre-registered replications. In addition, our participants were adolescent females and whether the same results would be found in males and children or older adults remains to be explored. Finally, we note two general limitations related to fMRI analyses. First, we suggest that the spatial distribution of our results is more in line with accounts of blushing that emphasize arousal and attention processes than with those emphasizing mentalizing. Such reverse inference should be interpreted with due care becauses the location of activation associated with mental processes as complex and pervasive as arousal, attention and mentalizing are not entirely distinct, which thereby limits our ability to compare the probability of these mental processes' involvement based on the pattern of activation [71]. Second, BOLD fMRI is not a direct measure of neural activity, and the true association strength between blushing and neural activity in the voxels that we identify thus remains difficult to estimate [72,73].

In summary, we provide evidence that blushing is associated with activity in the brain regions involved in emotional arousal and attention. This suggests that blushing may be triggered by emotional arousal and attentional engagement with self-relevant stimuli, without the involvement of mentalizing. These findings contribute to ongoing theoretical discussions regarding the nature of blushing and provide support for the idea that higher-order socio-cognitive processes may not be necessary for blushing to occur. The study informs the current debate on self-awareness more broadly, supporting recent approaches that increasingly recognize the importance of embodied experiences for self-awareness, and opens new avenues for research on self-awareness in infants and non-human animals.

## Data Availability

All summary data used for the figures as well as physiological and behavioural data can be found at Nikolic *et al.* [74]. Supplementary material is available online [75].
